# High preoperative bradykinin level is a risk factor for severe postoperative hypoxaemia in acute aortic dissection surgery

**DOI:** 10.1113/EP091054

**Published:** 2023-03-19

**Authors:** XinLiang Guan, Lei Li, JinZhang Li, WenJian Jiang, HaiYang Li, XiaoLong Wang, Lu Han, YuYong Liu, Ming Gong, HongJia Zhang

**Affiliations:** ^1^ Department of Cardiac Surgery, Beijing Aortic Disease Center, Beijing Anzhen Hospital Capital Medical University Beijing China; ^2^ Beijing Advanced Innovation Center for Big Data‐Based Precision Medicine Capital Medical University Beijing China; ^3^ Beijing Institute of Heart Lung and Blood Vessel Diseases Beijing China; ^4^ Beijing Laboratory for Cardiovascular Precision Medicine Key Laboratory of Medical Engineering for Cardiovascular Disease Beijing China

**Keywords:** acute aortic dissection, body mass index, bradykinin, hypoxaemia, risk factor

## Abstract

Severe hypoxaemia after cardiac surgery is associated with serious complications and a high risk of mortality. The purpose of this study is to investigate the independent risk factors of severe postoperative hypoxaemia in patients with acute Stanford type A aortic dissection. We collected 77 patients with acute Stanford type A aortic dissection who underwent surgical treatment. The primary outcome was severe postoperative hypoxaemia (PaO_2_/FiO_2_ ≤ 100 mmHg), and a multivariate logistic regression analysis was performed to assess the independent predictors of risk for this. A mixed‐effects analysis of variance model and a receiver operating characteristic (ROC) curve were generated to evaluate the predictive probabilities of risk factors for severe postoperative hypoxaemia. A total of 36.4% of patients developed severe postoperative hypoxaemia. The multivariate logistic regression analysis identified high preoperative bradykinin level (odds ratio (OR) = 55.918, *P* < 0.001) and increased body mass index (BMI; OR = 1.292, *P* = 0.032) as independent predictors of severe postoperative hypoxaemia in patients with acute Stanford type A aortic dissection. The mixed‐effect analysis of variance model and ROC curve indicated that high preoperative bradykinin level and BMI were significant predictors of severe postoperative hypoxaemia (area under the ROC curve = 0.834 and 0.764, respectively). High preoperative bradykinin levels and obesity were independent risk factors for severe postoperative hypoxaemia in patients with acute Stanford type A aortic dissection. For obese patients with high levels of bradykinin before surgery, clinicians should actively take measures to block bradykinin‐mediated inflammatory reactions.

## Abstract (highlights)

- **What is the central question of this study?**
  Hypoxaemia can lead to increased postoperative mortality in patients: what are the independent risk factors for severe hypoxaemia after acute Stanford type A aortic dissection?
- **What is the main finding and its importance?**
  Severe postoperative hypoxaemia was found in 36.4% of patients, and it was determined that high preoperative bradykinin levels and increased BMI were independent predictors of severe postoperative hypoxaemia in patients with acute Stanford type A aortic dissection. For obese patients with high preoperative bradykinin levels, more attention should be paid to preventing severe postoperative hypoxaemia.

## 1 INTRODUCTION

Hypoxaemia is one of the most urgent postoperative complications of cardiac surgery and is usually accompanied by poor prognosis, including prolonged ventilator support, increased hospital length of stay, increased intensive care unit (ICU) length of stay and higher perioperative mortality (Dunham et al., 2017; Sheng et al., 2022; Zhou et al., 2021). Despite improved perioperative management and surgical techniques, postoperative hypoxaemia has been reported to occur in 10–30% of patients after cardiac surgery (Filsoufi et al., 2008; Ranucci et al., 2014), and after aortic dissection surgery this figure is as high as 50% or more (Liu et al., 2017; Shen et al., 2018).

So far, there are no well‐defined and effective strategies for either prevention or treatment of postoperative hypoxaemia in the setting of cardiac surgery. Here we investigated the risk factors associated with severe hypoxaemia after acute Stanford type A aortic dissection surgery. Early identification of these risk factors will provide more time for surgeons to optimize the treatment of high‐risk patients. For these reasons, we conducted a retrospective cohort study to investigate the risk factors of severe postoperative hypoxaemia in patients with acute Stanford type A aortic dissection using a multivariate logistic regression model containing all known associated major perioperative predictors.

## 2 METHODS

### 2.1 Ethical approval

This study was supported by Beijing Anzhen Hospital and approved by the Hospital Ethics Committee in April 2018 (No. 2018004). The study conformed to the standards set by the *Declaration of Helsinki*.

### 2.2 Patient population

The Acute Aortic Syndrome Cooperation Network (AASCN) database is based on national key R & D projects and supported by the Ministry of Science and Technology of the People's Republic of China, the Ministry of Education, and the Beijing Municipal Commission of Science and Technology. Since the establishment of the database, more than 2500 patients with aortic syndrome and more than 11,000 specimens have been collected. Written informed consent was obtained for all data and specimens in the database from patients or their family members. In its scale the database ranks at the forefront in China, and the data for aortic dissection cover most of the people with aortic dissection in China (Guan et al., 2022; Li et al., 2021).

Preoperative lung disease or the use of angiotensin‐converting enzyme inhibitors would interfere with the results of this study, so this study excludes such people. Based on the AASCN database, we selected 77 patients with acute Stanford type A aortic dissection who underwent total arch replacement (TAR) combined with a frozen elephant trunk (FET) implant with cardiopulmonary bypass (CPB) in Beijing Anzhen Hospital from April 2020 to December 2021. All patients underwent urgent aortic arch surgery with or without aortic valve operations, involving moderate hypothermic circulatory arrest (HCA) at Beijing Anzhen Hospital. Patients who died intraoperatively or within 24 h postoperatively were excluded since no meaningful data were available for the evaluation of severe postoperative hypoxaemia.

### 2.3 Study design

In this single‐centre retrospective study, we analysed the preoperative characteristics, operative details and postoperative outcomes of 77 consecutive patients (54 men and 23 women; age range, 28–79 years; mean age, 48.2 ± 11.2 years) with acute Stanford type A aortic dissection who underwent TAR combined with a FET implant at Beijing Anzhen Hospital. Acute Stanford type A aortic dissection was diagnosed using enhanced computed tomography scanning, while aortic valve regurgitation was confirmed using echocardiography. All procedures were performed by the same surgery team. Trained staff collected detailed data from recruited patients from the electronic medical records at our medical centre. The hospital's Ethics Committee approved the study protocol, and consent was obtained from either the patients or their relatives.

The Berlin definition proposes three categories of hypoxaemia based on the degree of the condition: first, mild (200 mmHg < PaO_2_/FiO_2_ ≤ 300 mmHg); second, moderate (100 mmHg < PaO_2_/FiO_2_ ≤ 200 mmHg); and third, severe (PaO_2_/FiO_2_ ≤ 100 mmHg) (Ranieri et al., 2012). In the present study, according to the diagnostic criteria for acute respiratory distress syndrome (ARDS) established by the Berlin definition, severe postoperative hypoxaemia was defined as a PaO_2_/FiO_2_ ≤ 100 mmHg. The arterial blood gas and arterial partial pressure of oxygen to fraction inspired oxygen (PaO_2_/FiO_2_) were calculated for the perioperative period. It should be noted that the patients with severe postoperative hypoxaemia were identified within 72 h of receiving surgery, and we evaluated the condition within 72 h after each patient's arrival at the ICU. Seventy‐seven patients were divided into two groups according to postoperative PaO_2_/FiO_2_: first, a non‐severe hypoxaemia group (*n* = 49); and second, a severe hypoxaemia group (*n* = 28). In the event that more than one result was available for a given variable, the worst daily value was collected on days 0–3 of the perioperative period. Body mass index (BMI) values were recorded on admission using each patient's height and weight (BMI = weight (kg)/height (m)^2^). The primary endpoint of this study was to evaluate the incidence and risk factors of severe postoperative hypoxaemia in 77 patients with acute Stanford type A aortic dissection.

### 2.4 Surgical procedures

Standard anaesthetic management was used with endotracheal intubation. The procedure refers to TAR using a tetra‐furcate vascular graft in combination with the implantation of a FET into the descending aorta. Briefly, the procedure is performed with right axillary artery cannulation for CPB and antegrade cerebral perfusion (5–15 ml/kg/min) under HCA. After systemic heparinization (300 U/kg bodyweight and maintaining an activated clotting time longer than 480 s), CPB was established. During CPB, temperature‐adjusted flow rates of 2.5 L/min/m^2^ were used, and the mean arterial pressure was generally maintained between 50 and 70 mmHg. Our policy was to completely excise the primary tear according to the extent of disruption in each case. This procedure involves the implantation of a FET into the descending aorta, TAR with a four‐branched vascular graft, and a specific sequence for aortic reconstruction (proximal descending aorta, then left carotid artery, ascending aorta, left subclavian artery, and finally innominate artery). After completing distal anastomosis, CPB was reinstituted, and the patient was gradually rewarmed to a normal temperature after a 5‐min period of cold reperfusion for free radical washout. Proximal anastomosis was then performed.

### 2.5 Statistical analysis

The normality of the data distribution was tested using the Kolmogorov–Smirnov test. Data are expressed as the means ± standard deviation (SD) for continuous data with a normal distribution, as the median (25th and 75th percentiles) for continuous data with a non‐normal distribution, or numbers and percentages for categorical values. For comparison, one‐way analysis of variance or the Wilcoxon rank sum test was used for continuous variables, and the chi‐square test or Fisher's exact test was used for categorical variables. Logistic regression models were used to identify univariate and multivariate predictors for severe postoperative hypoxaemia. Univariate logistic regression analysis was used first to identify possible risk factors for severe postoperative hypoxaemia, and the multivariate model included variables that were found significant in the univariate analysis. A receiver operating characteristic (ROC) curve was generated to evaluate the predictive probabilities of risk factors for severe postoperative hypoxaemia using the area under the curve (AUC), 95% confidence interval (CI), specificity and sensitivity. The optimal cut‐off value was determined by calculating the Youden index of the ROC curve. In addition, to evaluate the effects of preoperative bradykinin (BK) level and BMI on postoperative levels of PaO_2_/FiO_2_, we created a mixed‐effects analysis of variance model. For all analyses, a probability value of less than 0.05 was considered statistically significant. All statistical analyses were performed using SPSS 18.0 (SPSS, Inc., Chicago, IL, USA).

## 3 RESULTS

Based on the AASCN database, 77 patients were enrolled in the study, with a mean age of 48.2 ± 11.2 years (28–79 years). The diagnosis of the enrolled population was based on aorta computed tomography angiography (CTA). On admission, a clotted false lumen was found on aorta CTA in 14 patients. Sixty‐one patients had dissections that extended below the diaphragm, while for 15, the dissection terminated above the diaphragm. There were no significant differences between the two groups with respect to preoperative imaging tests. Of these patients, 54 were male and 23 were female. The demographic, preoperative, intraoperative and postoperative clinical data associated with severe postoperative hypoxaemia are summarized in Table 1.

**TABLE 1:** Perioperative characteristics of patients with acute type A aortic dissection.

Characteristic	Non‐severe hypoxaemia (*n* = 49)	Severe hypoxaemia (*n* = 28)	*P*
Demographic data			
Age (years)	46.9 ± 11.7	50.5 ± 10.2	0.171
Male (*n* (%))	39 (79.6)	15 (53.6)	0.016
BMI (kg/m^2^)	24.8 ± 3.7	28.3 ± 3.6	<0.001
Medical history (*n* (%))			
Hypertension	36 (73.5)	23 (82.1)	0.387
Diabetes mellitus	0 (0.0)	2 (7.1)	0.129
Cerebrovascular disease	2 (4.1)	3 (10.7)	0.347
Coronary artery disease	2 (4.1)	3 (10.7)	0.347
Smoking history	20 (40.8)	12(42.9)	0.861
Marfan syndrome	2 (4.1)	3 (10.7)	0.347
Preoperative condition			
PaO_2_/FiO_2_	280.5 ± 87.1	258.2 ± 83.3	0.276
Aortic root size (mm)	42.1 ± 9.3	38.9 ± 4.7	0.050
Severe aortic regurgitation (*n* (%))	27 (55.1)	10 (35.7)	0.101
Ascend aorta size (mm)	45.9 ± 7.7	44.8 ± 6.3	0.525
Left ventricular ejection fraction (%)	61.7 ± 6.1	63.0 ± 6.6	0.412
Haemopericardium (*n* (%))	5 (10.2)	8 (28.6)	0.057
Operation details			
The duration of operation (h)	7.6 ± 1.6	8.6 ± 1.6	0.007
CPB time (min)	197.4 ± 48.7	229.3 ± 61.5	0.014
Aortic cross clamp time (min)	120.4 ± 49.5	130.6 ± 35.4	0.344
The duration of HCA (min)	27.8 ± 10.5	28.0 ± 7.5	0.955
Nasopharyngeal temperature (°C)	23.2 ± 2.1	22.3 ± 1.5	0.049
Rectal temperature (°C)	25.6 ± 2.5	25.4 ± 2.3	0.683
Intraoperative blood loss (ml)	1342.9 ± 482.2	1435.7 ± 662.3	0.481
Intraoperative amount of plasma (ml)	400 (0, 400)	400 (0, 800)	0.193
Intraoperative amount of PRBCs (ml)	150 (0, 600)	600 (0, 600)	0.020
Postoperative outcomes			
Length of ICU stay (days)	1.5 (1.0, 3.8)	7.0 (3.6, 13.5)	<0.001
Length of hospital stay (days)	14 (10, 20)	16 (12, 18.8)	0.679
In‐hospital mortality (*n* (%))	2 (4.1)	6 (21.4)	0.024
Reoperation for bleeding (*n* (%))	2 (4.1)	4 (14.3)	0.182
Postoperative dialysis (*n* (%))	4 (8.2)	12 (42.9)	<0.001
Low cardiac output syndrome (*n* (%))	1 (2.0)	4 (14.3)	0.056
Sepsis (*n* (%))	6 (12.2)	6 (21.4)	0.336
Paraplegia (*n* (%))	1 (2.0)	1 (3.6)	1.000
Cerebral infarction or bleeding (*n* (%))	0 (0.0)	7 (25.0)	<0.001

Values are means ± SD, *n* (%) or median (IQR). Abbreviations: BMI, body mass index; CPB, cardiopulmonary bypass; HCA, hypothermic circulatory arrest; ICU, intensive care unit; PRBCs, packed red blood cells.

### 3.1 Preoperative details

As shown in Table 1, which provides an overview of patient characteristics and perioperative variables, the incidence of severe postoperative hypoxaemia in our patient population was 36.4% (28/77). In our study, all patients breathed spontaneously with nasal prongs or face masks with inhaled oxygen at 5–8 litres/min prior to surgical intervention. In order to gain insight into the factors associated with severe postoperative hypoxaemia, the patient population was divided into two groups based on each individual's postoperative PaO_2_/FiO_2_. Patients with postoperative PaO_2_/FiO_2_ ≤ 100 mmHg were classified into the severe hypoxaemia group, and the other patients were classified into the non‐severe hypoxaemia group.

Among the preoperative characteristics, Table 1 shows that BMI values were higher for the severe hypoxaemia group when compared to the non‐severe hypoxaemia group (*P* < 0.001). Notably, no significant differences in medical history were observed between the two groups. Preoperative laboratory tests between the two groups are summarized in Table 2. Furthermore, white blood cells, neutrophil ratio and bradykinin (BK) were higher in the severe hypoxaemia group compared to patients with non‐severe hypoxaemia (*P* = 0.005, *P* < 0.001 and *P* < 0.001, respectively).

**TABLE 2:** Comparison of laboratory tests on admission between two groups.

Characteristics	Non‐severe hypoxaemia (*n* = 49)	Severe hypoxaemia (*n* = 28)	*P*
sCr (μmol/l)	83.1 ± 30.6	92.5 ± 33.4	0.213
Troponin I (ng/ml)	0.02 (0.00, 0.06)	0.03 (0.00, 0.08)	0.668
White blood cells (×10^3^ cells/μl)	10.0 ± 3.6	12.5 ± 3.4	0.005
Neutrophil ratio	76.7 ± 9.2	82.9 ± 5.8	<0.001
Haemoglobin (g/dl)	138.3 ± 19.2	134.6 ± 16.8	0.409
Fibrinogen (g/l)	3.6 ± 1.6	3.6 ± 1.9	0.884
FDP (μg/ml)	12.0 (6.6, 27.9)	20.7 (14.2, 60.4)	0.014
D‐dimer (μg/ml)	1039.5 (590.5, 2373.0)	2410.0 (896.3, 6078.5)	0.013
TNF‐α (pg/ml)	46.0 ± 9.9	47.5 ± 12.2	0.567
IL‐1 (pg/ml)	45.8 ± 10.0	44.5 ± 10.0	0.602
IL‐2 (pg/ml)	898.5 ± 81.3	899.6 ± 81.0	0.952
IL‐6 (pg/ml)	207.9 ± 30.7	212.8 ± 48.2	0.586
BK (ng/ml)	3.2 ± 0.4	3.5 ± 0.4	<0.001
PK (pg/ml)	487.2 ± 34.6	475.8 ± 34.7	0.173
Kallikrein	406.7 ± 18.3	408.3 ± 25.8	0.752
CRP	4.3 ± 1.3	4.2 ± 1.2	0.613

Values are means ± SD or median (interquartile range). Abbreviations: BK, bradykinin; CRP, C‐reaction protein; FDP, fibrinogen degradation products; IL, interleukin; PaO_2_/FiO_2_, arterial partial pressure of oxygen to fraction of inspired oxygen; PK, plasma kallikrein; sCr, serum creatinine; TNF‐α, tumour necrosis factor‐α.

### 3.2 Operation details

Operation details are presented in Table 1. No significant differences were observed between the two groups in terms of aortic occlusion and HCA duration. Patients with severe hypoxia had longer operation time and CPB time (*P* = 0.007 and *P* = 0.014, respectively). Nasopharyngeal temperature and rectal temperature values were not significantly different between two groups. Moreover, with regard to intraoperative blood loss and intraoperative amount of plasma or packed red blood cells, there were also no significant differences between the two groups.

### 3.3 Postoperative details

Postoperative clinical details are summarized in Table 1. Overall, in‐hospital mortality was 10.4% (8/77) for patients with acute Stanford type A aortic dissection. In the patient population, in‐hospital mortality was 21.4% (*n* = 6) for patients suffering from severe hypoxaemia, while it was 4.1% (*n* = 2) for non‐severe hypoxaemia patients (*P* = 0.024). Among the postoperative characteristics, the postoperative clinical outcomes were complicated in patients with severe hypoxaemia, with a higher rate of complications, such as longer ICU stay, postoperative dialysis, and cerebral infarction or bleeding (*P* < 0.001, *P* < 0.001 and *P* < 0.001, respectively).

### 3.4 Multivariate logistic regression analysis associated with severe postoperative hypoxaemia

The risk factors for severe postoperative hypoxaemia found using multivariate logistic regression analysis are shown in Table 3. In a primary model, all preoperative risk factors and intraoperative parameters of recognized clinical significance were included. Significant differences were found in univariate logistic regression analysis between non‐severe hypoxaemia patients and severe hypoxaemia patients in terms of increased BMI, the duration of operation, CPB time, white blood cells, neutrophil ratio and BK. High preoperative BK level (OR, 55.918 (95% CI: 4.273, 731.806); *P* < 0.001) and increased BMI (OR, 1.292 (95% CI: 1.023, 1.632); *P* = 0.032) were still identified as independent risk factors for severe postoperative hypoxaemia in the multivariate logistic regression analysis.

**TABLE 3:** Risk factors for postoperative severe hypoxaemia in multivariate logistic regression analysis in patients with acute Stanford type A aortic dissection.

Clinical variable	OR (95% CI)	*P*
BMI (kg/m^2^)	1.292 (1.023, 1.632)	0.032
The duration of operation (h)	1.276 (0.720, 2.260)	0.404
CPB time (min)	1.008 (0.989, 1.027)	0.419
Lactate (mmol/L)	1.785 (0.851, 3.741)	0.125
White blood cell (×10^3^ cells/μl)	1.193 (0.908, 1.567)	0.204
Neutrophil ratio	1.107 (0.993, 1.233)	0.066
Preoperative BK level	55.918 (4.273, 731.806)	<0.001

Abbreviations: BMI, body mass index; CI, confidence interval; CPB, cardiopulmonary bypass; OR, odds ratio.

### 3.5 Risk prediction for severe postoperative hypoxaemia

**FIGURE 1:**
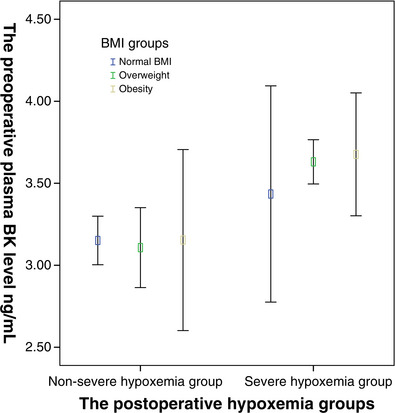
The mixed‐effects analysis of variance model showed that the preoperative BK level and BMI were associated with incidence of severe postoperative hypoxaemia in patients with acute Stanford type A aortic dissection (*P* < 0.001).

A mixed‐effects analysis of variance model was used to evaluate the impact of preoperative BK level and BMI on severe postoperative hypoxaemia (Figure 1). Figure 1 shows that compared with the non‐severe hypoxaemia group, the severe hypoxaemia group had a higher level of preoperative BK. At the same time, in the severe hypoxaemia group, overweight and obesity groups showed higher BK levels (*P* < 0.001).

**FIGURE 2:**
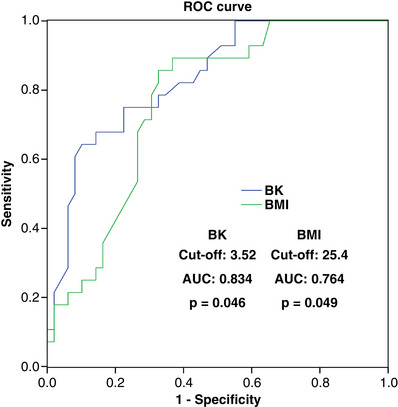
High preoperative BK level and increased BMI as predictive value of risk factor for severe postoperative hypoxaemia in patients with acute Stanford type A aortic dissection by ROC curve analysis.

ROC curves were used to determine the predictive and cut‐off values of risk factors for severe postoperative hypoxaemia. As shown in Figure 2, high preoperative BK level (cut‐off, 3.52; AUC, 0.834; sensitivity, 67.9%; specificity, 85.7%; *P* = 0.046) and BMI (cut‐off, 25.4; AUC, 0.764; sensitivity, 85.7%; specificity, 63.3%; *P* = 0.049) were significant predictors of severe postoperative hypoxaemia.

## 4 DISCUSSION

Based on the AASCN database, 77 patients with type A aortic dissection who underwent emergency surgery in Beijing Anzhen Hospital from April 2020 to December 2021 were included in the study. The average age of the population in this study was 48.2 ± 11.2 years old. Compared with the European and American races, the age of onset of type A aortic dissection in the Asian population is lower (Wang et al., 2014). Compared to other elective cardiac surgical procedures (Filsoufi et al., 2008; Ranucci et al., 2014), in recent years postoperative hypoxaemia has been one of the most common life‐threatening complications after acute Stanford type A aortic dissection surgery, accompanied by an increased risk of morbidity or mortality and higher hospital costs (Raijmakers et al., 1997). In order to improve the clinical prognosis of acute Stanford A aortic dissection, it is particularly important to study the risk factors of severe postoperative hypoxaemia (PaO_2_/FiO_2_) ≤ 100 mmHg). In our study, the incidence of severe postoperative hypoxaemia was identified as 36.4% (28/77) for acute Stanford type A dissection surgery. At present, the potential mechanism of severe hypoxaemia after acute Stanford A dissection is still unclear. Thus, further understanding of the risk factors and mechanism of severe hypoxaemia after acute Stanford type A dissection surgery is crucial to the design of preventative strategies.

In order to further understand the factors related to severe postoperative hypoxaemia, the research team assigned patients with PaO_2_/FiO_2_ ≤ 100 mmHg to a severe hypoxaemia group, while other patients were assigned to a non‐severe hypoxaemia group. The comparison of in‐hospital mortality between the two groups (21.4% vs. 4.1%, *P* = 0.024) suggests that severe hypoxaemia is a high‐risk factor for in‐hospital mortality in patients with acute Stanford type A dissection. The study analysis found postoperative complications of patients with severe hypoxaemia, such as longer hospitalization in ICU (*P* < 0.001), higher postoperative dialysis and more cerebrovascular adverse events (*P* < 0.001 and *P* < 0.001, respectively). This suggests that it is necessary to understand the risk factors and mechanisms of severe hypoxaemia after acute Stanford A dissection.

The study found that there was a significant difference between the two groups in the indicators of the inflammatory system detected in the laboratory before operation. The preoperative leukocytes, neutrophil ratio and bradykinin (BK) in the severe hypoxaemia group were significantly higher than those in the non‐severe hypoxaemia group (*P* = 0.005, *P* < 0.001, and *P* < 0.001, respectively). Previous studies have found that leukocytes and neutrophils are highly correlated with decreased pulmonary function (Sefik et al., 2022; Zhang et al., 2021), but there are few studies on hypoxaemia after aortic dissection. Our study found that BK, as the core molecule of the inflammatory system, was significantly increased in people with severe hypoxaemia. This suggests that BK is more likely to participate in the occurrence of severe hypoxaemia after acute Stanford A dissection. We conducted a multiple logistic regression analysis on the above risk factors. We found that high preoperative BK levels and increased BMI were independent risk factors for severe hypoxaemia after acute Stanford A dissection.

The kallikrein–kinin system is a zymogen system that after activation leads to the release of the nonapeptide BK. Kallikreins are serine proteases and can be divided into plasma kallikrein and tissue kallikreins. Both the plasma and the tissue kallikreins release the vasoactive peptide BK (Marceau et al., 2018), a linear nonapeptide formed by the proteolytic activity of kallikrein on kininogens. BK binding to the BK receptor B2 (B2R) on endothelial cells has strong vasopermeability and vasodilatory effects, which can lead to the relaxation of vascular smooth muscle, increased vascular permeability, capillary leakage and thus angio‐oedema (van de Veerdonk et al., 2020). Past research has demonstrated that increased BK might promote vasodilatation and increases vascular permeability and inflammation, mainly through the constitutively expressed B2R (Maurer et al., 2011). On one hand, a randomized, placebo‐controlled clinical trial of a B2R antagonist demonstrated some effect on survival in patients with systemic inflammatory response syndrome (Fein et al., 1997). On the other hand, studies in animals suggested that B2R antagonism inhibited reperfusion‐induced increases in vascular permeability and vasodilatation (Souza et al., 2003). We believe that the BK‐induced inflammatory cascade reaction may be an important reason for severe hypoxaemia in patients with acute Stanford A aortic dissection after surgery. In the acute Stanford A aortic dissection, the sudden rupture of the intima and the diffusion of the dissection to the middle layer are related to the activation of the systemic inflammatory response. Inflammatory reactions and oxidative stress play a key role in damaging alveolar epithelium and capillary endothelial cells (Gao et al., 2019).

Higher BMI and obesity have been widely reported to be independently associated with the development of postoperative hypoxaemia and severe postoperative hypoxaemia in patients with acute Stanford type A aortic dissection, which is in agreement with the result of our study (Zhang et al., 2022). The obvious decrease in lung compliance, excessive growth of the upper respiratory tract and respiratory resistance in obese patients may be associated with breathing difficulties and resultant hypoxaemia (Gong et al., 2019). Moreover, because adipose tissue can release numerous peptides and cytokines into the blood circulation (Kawai et al., 2021), subclinical chronic inflammation and oxidative stress in the pathogenesis of acute aortic dissection may play a synergistic role in pulmonary complications for obese individuals. Consequently, obesity was considered as an independent high‐risk factor of postoperative pulmonary complications (de Lima et al., 2016; Quante et al., 2015) and improved intra‐ or postoperative ventilation strategies will be crucial for such populations (Ball et al., 2018). The inflammatory response and oxidative stress may be involved in the process of lung injury of acute Stanford type A aortic dissection caused by obesity, which has provided new ideas for the treatment (Gong et al., 2019).

The mixed‐ effects analysis of variance model suggested that the preoperative increased BMI combined with high preoperative bradykinin level population had a higher probability of postoperative hypoxaemia. For the above population, clinicians should pay close attention to pulmonary function during the perioperative period and actively give medical intervention. Preoperative increased BMI (>25.4) and high preoperative BK level (>3.52) can effectively predict the occurrence of postoperative hypoxaemia in patients with acute Stanford type A aortic dissection. Hypoxaemia is one of the most common life‐threatening complications after acute Stanford A aortic dissection, accompanied by an increase in morbidity or mortality and higher hospital costs. We hope that this study can effectively improve the prognosis of patients with acute Stanford A aortic dissection, reduce mortality and reduce social burden. Of course, the clinical application of the above results still needs more in‐depth and rigorous research.

### 4.1 Study limitations

Several potential limitations of the present study should be discussed. First, the patient population collected for investigation was relatively small and only in a single institution, which limited the applicability of our findings to other settings. Second, the influence of factors such as the experience of the individual surgeon and institutional philosophy on the decision made regarding the treatment was not considered for this analysis. Finally, patients who died within 24 h may possibly have had hypoxaemia. Since such patients were not used to evaluate the data related to postoperative hypoxaemia, they were not included in the study.

### 4.2 Conclusions

In conclusion, we found that two independent risk factors for severe postoperative hypoxaemia in patients undergoing acute Stanford type A aortic dissection were high preoperative BK level and BMI. The results suggested that high preoperative BK level was the preferred marker for predicting severe postoperative hypoxaemia, especially in obese patients. Therefore, appropriate preventive measures ought to be taken to minimize the incidence of severe postoperative hypoxaemia in obese patients with high preoperative BK level. These measures may include reducing BK‐mediated inflammation response and local pulmonary angio‐oedema related to severe postoperative hypertension. At the same time, the perioperative respiratory system adjustment for such patients should be more active, such as ventilator adjustment and early respiratory muscle physical therapy.

## AUTHOR CONTRIBUTIONS

Conception or design of the work: Ming Gong and HongJia Zhang. Acquisition, analysis or interpretation of data for the work: XinLiang Guan, Lei Li, JinZhang Li, WenJian Jiang, HaiYang Li, XiaoLong Wang, Lu Han and YuYong Liu. Drafting of the work or revising it critically for important intellectual content: XinLiang Guan, Lei Li, JinZhang Li, WenJian Jiang, Ming Gong and HongJia Zhang. All authors approved the final version of the manuscript. All authors agree to be accountable for all aspects of the work in ensuring that questions related to the accuracy or integrity of any part of the work are appropriately investigated and resolved. All persons designated as authors qualify for authorship, and all those who qualify for authorship are listed.

## CONFLICT OF INTEREST

The authors have nothing to disclose with regard to commercial support.

## Supporting information


Statistical Summary Document

## Data Availability

The datasets analysed for this study are available from the corresponding author on reasonable request.
